# Improving Adherence to Adjuvant Hormonal Therapy Among Disadvantaged Women Diagnosed with Breast Cancer in South Carolina: Proposal for a Multimethod Study

**DOI:** 10.2196/17742

**Published:** 2020-09-03

**Authors:** Tisha M Felder, Sue P Heiney, James R Hebert, Daniela B Friedman, Ronit Elk, Regina Franco, Lucy Gansauer, Barbara Christensen, Marvella E Ford

**Affiliations:** 1 College of Nursing University of South Carolina Columbia, SC United States; 2 Cancer Prevention & Control Program Arnold School of Public Health University of South Carolina Columbia, SC United States; 3 Department of Epidemiology and Biostatistics Arnold School of Public Health University of South Carolina Columbia, SC United States; 4 Department of Health Promotion, Education, and Behavior Arnold School of Public Health University of South Carolina Columbia, SC United States; 5 Office for the Study of Aging Arnold School of Public Health University of South Carolina Columbia, SC United States; 6 Center for Palliative and Supportive Care Division of Geriatrics, Gerontology, and Palliative Care University of Alabama-Birmingham School of Medicine Birmingham, AL United States; 7 Center for Integrative Oncology & Survivorship Cancer Institute Prisma Health Greenville, SC United States; 8 Gibbs Cancer Center & Research Institute Spartanburg Regional Healthcare System Spartanburg, SC United States; 9 Department of Public Health Sciences College of Medicine Medical University of South Carolina Charleston, SC United States; 10 Population Sciences and Cancer Disparities Hollings Cancer Center Medical University of South Carolina Charleston, SC United States; 11 Office of Community Outreach and Engagement Hollings Cancer Center Medical University of South Carolina Charleston, SC United States; 12 SmartState Endowed Chair, Cancer Disparities Research Medical University of South Carolina and South Carolina State University Charleston, SC United States

**Keywords:** breast neoplasms, medicaid, medication adherence, vulnerable populations, hormonal therapy, endocrine therapy, qualitative methods

## Abstract

**Background:**

Current clinical guidelines recommend that hormone receptor–positive breast cancer survivors take adjuvant hormonal therapy (AHT) for 5 to 10 years, following the end of definitive treatment. However, fewer than half of patients adhere to the guidelines, and suboptimal adherence to AHT is associated with an increased risk of breast cancer mortality. Research has extensively documented sociodemographic and disease-specific factors associated with adherence to AHT, but very little evidence exists on behavioral factors (eg, knowledge, patient-provider communication) that can be modified and targeted by interventions.

**Objective:**

The goal of this study is to develop and test a theory-based, multilevel intervention to improve adherence to AHT among breast cancer survivors from racially and socioeconomically disadvantaged backgrounds (eg, Medicaid-insured). The specific aims are to (1) explore multilevel (eg, patient, health care system) factors that influence adherence to AHT; (2) develop a theory-based, multilevel intervention to improve adherence to AHT; and (3) pilot test and evaluate the intervention developed in Aim 2.

**Methods:**

For Aim 1, we will recruit breast cancer survivors and health care professionals to participate in semistructured interviews to gain their perspectives about barriers and facilitators to AHT use. We will conduct a directed content analysis of the Aim 1 qualitative interview data. For Aim 2, we will integrate Aim 1 findings and current literature into the design of a multilevel intervention using an Intervention Mapping approach. For Aim 3, we will recruit Medicaid-insured breast cancer survivors to assess the feasibility of the pilot intervention.

**Results:**

From May 2016 to July 2018, we completed interviews with 19 breast cancer survivors and 23 health care professionals in South Carolina. We will conduct a directed content analysis of the qualitative interview data. Results from this analysis will be used, in combination with current literature, to design (Aim 2) and pilot test a theory-based multilevel intervention (Aim 3) in Summer 2021. Results of the pilot are expected for Fall 2021.

**Conclusions:**

This study will provide a deeper understanding of how to improve adherence to AHT, using a novel and multilevel approach, among socioeconomically disadvantaged breast cancer survivors who often experience disproportionate breast cancer mortality.

**International Registered Report Identifier (IRRID):**

DERR1-10.2196/17742

## Introduction

### Background

Adjuvant hormonal therapy (AHT) has been shown to significantly reduce recurrence and mortality rates among women diagnosed with hormone receptor–positive (HR+) breast cancer [1]. Current clinical guidelines for women diagnosed with early stage (stages I-III) HR+ breast cancer recommend up to 10 years of AHT with tamoxifen or an aromatase inhibitor (AI), following surgery, chemotherapy and/or radiation, as indicated. [2,3]. However, although AHT is considered the standard of care for breast cancer, reports show that only about 50% of women complete treatment as recommended [4,5].

Systematic reviews show that sociodemographic factors, such as race and age, are associated with treatment adherence. In contrast, there is limited evidence about psychosocial or behavioral factors that could be targeted with interventions [4,6,7]. Additionally, previous studies that have examined modifiable factors suggest that negative beliefs about AHT, limited social support, low decisional balance scores and poor patient-provider communication are all negatively associated with therapy adherence [8-12]. Early discontinuation of and poor adherence to AHT have been significantly associated with disease progression and increased morality rates [5,13].

The problem of suboptimal adherence to AHT is important for three key reasons. First, roughly 75% of diagnosed breast cancers are HR+ [14], meaning that adherence to AHT is critical for extending the survival of the majority of breast cancer survivors. Second, studies show that differences in treatment, including AHT use, significantly contribute to persistent racial disparities observed in breast cancer mortality rates between Black and White women [15,16]. This disparity increases for women who are socioeconomically disadvantaged [17,18]. Third, rates of adherence to AHT are lowest among financially disadvantaged populations versus other population–based groups. For example, only 58% of Medicaid-insured women adhered to AHT in the first year [19], compared to 80%-85% in privately insured populations [20,21]. Hershman et al [22] recently found that low annual household income (<US $40,000) significantly decreased the odds of Black women adhering to AHT compared to White women. Therefore, the improvement of adherence to AHT among socioeconomically disadvantaged Black women with breast cancer is critical to increase their survival rate as well as to close the gap on racial disparities in breast cancer treatment.

### Project Goal and Innovation

The overall goal of this study is to improve adherence to AHT among breast cancer survivors from racially and socioeconomically disadvantaged backgrounds. The specific aims of this study are as follows: (1) explore patient, health care system, and structural factors that may influence adherence to AHT; (2) develop a theory-based, multilevel intervention program to improve adherence to AHT; and (3) pilot and evaluate the intervention program designed in Aim 2.

This project will incorporate principles of health communication theory into the development and testing of the proposed multilevel intervention program. The field of health communication has been nationally prioritized as a strategy to improve individual and population health [23]. Health communications reflect an ecological perspective which posits that individual health beliefs and behaviors are influenced by the broader social environment. Thus, effective public health communication can improve health behaviors by using strategies that consider multiple levels of influence (eg, intrapersonal, interpersonal, community) [24]. The use of health communication theory in this study represents a novel approach to fill a gap in the literature on how to apply theory in multilevel interventions. Furthermore, the application of theory will yield a practical framework for how to use health communication theoretical constructs to determine appropriate communication strategies (eg, knowledge, attitudes) that will target factors at multiple levels known to influence AHT adherence, and how to test them in a multilevel intervention.

## Methods

### Study Overview

This project received funding from the National Cancer Institute of the National Institutes of Health (2015-2020). The proposed research will address concepts proposed by the World Health Organization’s (WHO) Multidimensional Adherence Model Framework [25] and the Multi-level Context of Cancer Care Model [26] (Table 1). This adapted framework shows how factors are nested within multiple levels to influence AHT adherence and can be useful for designing and targeting multilevel interventions. The formative, in-depth, semistructured interviews (Aim 1, Years 1-2) will constitute the basis for the development of culturally appropriate messaging and content of the multilevel intervention (Aim 2; Year 2) to be pilot-tested and evaluated in Year 3 (Aim 3).

**Table 1:** Modifiable factors at multiple levels that influence adherence to adjuvant hormonal therapy [27-29].

Level and factors		Related factors	Potential interventions
Patient		Psychosocial factors	Education + behavioral support
		Knowledge, beliefs, attitudes	Case management
		Illness perceptions	Pharmacist-led, multicomponent intervention
Family/social support		Family dynamics	Family/dyadic education
		Friends, network support	
		AHT recommendations	Enhance skill mix/competencies of care team
Health care system/health care team		AHT fill interval	Reminders
		Communication preferences/styles	Education on effective patient-centered communication + behavior management
		Perceptions/bias	
		Cost of AHT	Reduced out-of-pocket expenses
Policy/structural/cultural		National/state/local policy	Public health insurance coverage
		Distance to health/pharmacy services	

### Aim 1. Explore Patient, Health Care System, and Structural Factors That May Influence Adherence to AHT

#### Study Design

Study Aim 1 will explore patient, health care system, and structural factors that influence adherence to AHT (Years 1-2). To achieve this aim, in-depth, semistructured qualitative interviews will be conducted with health care professionals who work in oncology settings and with breast cancer survivors. For purposes of this research, we define a “breast cancer survivor” as anyone who has been diagnosed with invasive breast cancer.

#### Health Care Professional Recruitment and Eligibility

Health care professionals will be recruited to participate in qualitative interviews. The study will target health professionals from key networks and organizations in South Carolina, including professional organizations (eg, oncology nursing, oncology social work), cancer centers and South Carolina’s cancer coalition and department of health. The primary method of recruitment will be through email invitation letters sent by the study’s principal investigator (PI).The invitation letter will describe the study aims, study procedures, funding source, and institutional review board approval protocol information. Interested health care professionals will be able to contact the study PI directly by telephone or email. The PI will schedule an interview at a time and location convenient to the potential participants to determine their eligibility for the study. Eligibility requirements will include (1) being employed as a health care professional (ie, physicians, nurses, pharmacists, social workers, patient navigators); (2) currently working with breast cancer patients, for example in an oncology setting, such as a cancer center or hospital; and (3) age ≥ 21 years.

#### Health Care Professional Data Collection

Before the interview, health care professionals will be asked to complete a brief questionnaire about their personal (eg, gender, age) and professional (eg, current job title, type of health care professional, health care setting) information. The PI will conduct all interviews using a guide developed by the study team. Key topics include: (1) major barriers to AHT adherence; (2) organizational resources to support posttreatment (eg, surgery, chemotherapy) of breast cancer survivors; (3) communication among health care team members; (4) communication with patients and their families; and (5) organizational strategies for addressing problems with AHT adherence. Participants will receive a US $50 cash stipend for their time and effort. At the end of the interview, participants will be asked for their contact information if they are willing to participate in a follow-up feedback session, which will serve to inform the development of a subsequent intervention. All interviews will be digitally recorded and field notes will be taken.

#### Breast Cancer Survivor Recruitment and Eligibility

Breast cancer survivors will be recruited from the South Carolina Oncology Associates (SCOA) and the Gibbs Cancer Center and Research Institute (GCCRI). Eligibility requirements include: (1) age≥21 years; (2) diagnosed with HR+, invasive breast cancer; (3) having medical records that confirm the prescription of any hormonal treatment (anastrozole, exemestane, letrozole, tamoxifen) at any point after diagnosis; (4) eligible for or enrolled in South Carolina Medicaid program; and (5) being able to speak and read in English.

To recruit participants, we will apply a recruitment model developed by Heiney and colleagues [30], which combine social marketing with relationship building. The PI and a research assistant will work with SCOA and GCCRI staff to identify participants who meet the study eligibility criteria using electronic medical records. SCOA patients will be mailed a personal invitation letter from the PI explaining the goals of the study, including a Frequently Asked Questions document that addresses potential concerns of research participants. The letter will include a phone number to call if the participant prefers not to be contacted. Within 3-5 days after the recruitment letter is mailed, the PI or research assistant will contact the participant by phone to provide them with an overview of the study. If the participant expresses interest in the study, the PI or research assistant will verify the participant’s study eligibility. If eligibility is met, an interview appointment will be scheduled at a time and location convenient to the participant. If a participant is not eligible, the PI or research assistant will let the participant know that they did not meet the study criteria and will thank them for their interest in participating.

At GCCRI, designated staff will contact potentially eligible breast cancer survivors by phone and provide them with an overview of the study. If the participant shows interest in the study, GCCRI staff will then ensure the participant meets study eligibility. If eligibility is met, GCCRI staff will request verbal consent by phone and set up an interview appointment at GCCRI. Interested and eligible participants will be mailed an “Appointment Form” confirming the date, time, and location of the interview; a copy of the informed consent; and the Frequently Asked Questions document.

#### Breast Cancer Survivor Data Collection

Interviews with participants recruited from SCOA will be scheduled and conducted at a location (eg, home, library) and time convenient to the participant, while GCCRI participant interviews will be scheduled in person and onsite at GCCRI. Before the interviews, the following informed consent process will be conducted: a research team member (PI or research assistant) will read aloud a consent form that will inform the participant about the following components of the study: title of the research project, the identity of the project PIs and their respective contact information, an introduction about the study, the aims of the study, reason for the project, a summary of why the individual has been asked to participate, a description of the study procedures (ie, audiotaping of interviews), an outline of any risks and/or benefits, an overview of how confidentiality will be maintained, a discussion of any benefits, and a method of securing additional information or asking questions. After the consent form is read aloud and the participant agrees to participate in the study, the research team member and participant will sign and date the form. Participants will be given a copy of the form for their records.

Following consent, participants will be asked to complete a brief information form about their personal information (eg, age, race, marital status), hormonal therapy (eg, type of drug, daily use), and type of AHT resources used and preferred. The research team will conduct all interviews using a semistructured interview guide. Breast cancer survivors will be asked to describe their experiences with AHT, including experiences communicating with health care professionals about AHT, and strategies used for addressing AHT nonadherence. All participants will be given a US $50 cash stipend for their time and effort. At the end of the interview, participants will be asked if they are willing to participate in the intervention development phase of this study (Aim 2).

#### Data Analysis of Interviews

All interviews will be recorded and transcribed verbatim by a professional transcription service. Each participant’s audio file and transcript will be assigned the same unique identifier as their corresponding brief questionnaire. Quality control checks will be conducted while reviewing the transcripts to ensure that no personal identifiers are used. Audiotaped transcripts and field notes will be used for data analyses. Transcripts will be independently read and reviewed to check for accuracy and authenticity.

To identify potential modifiable factors and future intervention targets for Aim 2, Assarroudi et al’s [31] qualitative content analysis approach will be used to analyze interview transcripts. The PI will develop a preliminary codebook of main categories and subcategories derived from the WHO Multidimensional Adherence Model Framework [25] and Multilevel Context of Cancer Care [26]. The PI will work with other team members to discuss the codebook as well as the final main categories and subcategories and their related meanings. The PI and two other research team members will independently read and code a selection of transcripts using the preliminary codebook. Following this initial coding, all team members will meet, compare coding results, and discuss differences in coding interpretations and concerns with the preliminary codebook. The PI will revise the codebook as needed. Once the codebook is finalized, a research assistant who is knowledgeable in the study topic area will, independently, code all the transcripts, and the PI will code a random sample of the transcripts. The PI and research assistant will then meet and discuss coded transcripts, reach a consensus among any discrepancies and calculate intercoder agreement [32].

### Aim 2. Develop a Theory-based, Multi-level Intervention Program to Improve Adherence to AHT

#### Overview of Intervention Development Via Data Integration/synthesis and Intervention Mapping

Multiple sources will be used for the development of a theory-based, multilevel intervention program, including findings from Aim 1, current scientific literature, insights from participant feedback sessions (described below), and expertise of the project mentoring team. After analyzing the qualitative data from the breast cancer survivor and health care professional interviews, we will develop a modified, data-informed logic model of potential interventions aimed at health care professionals and their organizations, and survivors and their family/social support. Logic models, representing an outcomes hierarchy, will assist with a broad understanding of the components of each potential intervention [33]. Potential intervention strategies are shown in Table 1 [27-29]. The research team will systematically review and compare findings from the survivor and health care professional qualitative interviews and quantitative questionnaire data. Of interest is not only the variation within the survivor and health care professional groups, but also the variation between the survivor and health care professional reports. We will, therefore, identify themes that occur within and across groups. We will employ a logic model based on the PRECEDE approach [34] to illustrate survivors’ experiences with AHT and how knowledge, perceptions and attitudes at different levels impact their ability to adhere to AHT. We will apply appropriate multi-level theories to refine the intervention strategies and messages [35-37].

#### Validation of Formative Data and Intervention Design Through Participant Feedback Session

From the group of participants that agreed to participate in a follow-up feedback session, we will select a purposive sample of breast cancer survivors (n=5; and their designated family member/friend, n=5) and health care professionals (n=5), based on their brief questionnaire and interview responses. We will host two, 1 to 2 hours feedback sessions: one for breast cancer survivors and their family member/social support, and another one for health care professionals. The feedback sessions will provide participants with the opportunity to judge the accuracy and credibility of the qualitative research findings, as well as to provide alternative interpretations and explanations when necessary [38]. We will also discuss any differences of opinion that may appear between the survivor and health care professional interviews to reach a resolution. Participants will receive US $50 and a light meal for their time and effort upon completion of the session. The project mentoring team will provide guidance on how to refine the intervention design based on the data from the feedback sessions and resolve any data discrepancies.

## Results

The institutional review boards of the University of South Carolina, Prisma Health (formerly Greenville Health System) and Spartanburg Regional Hospital System reviewed the study protocols for Aim 1 and determined that they were exempt from human research subject regulations. Qualitative data collection for Aim 1 began in May 2016 and has been completed as of July 2018. A total of 19 breast cancer survivors and 23 health care professionals have been interviewed. Qualitative data analysis will be completed by December 2020. Results from this analysis will be used, in addition to current literature, to design (Aim 2) and pilot test a theory-based multilevel intervention (Aim 3) by Summer 2021. Multiple sources will be used for the development of a theory-based, multilevel intervention, including findings from Aim 1, current scientific literature, insights from participant feedback sessions (Aim 2), expertise of the research team, and trainings from the Multilevel Intervention Training Institute at the National Cancer Institute.

## Discussion

Findings from this research will help fill a gap in our current understanding of underlying modifiable factors that influence adherence to AHT, particularly among understudied disparate populations [4]. To our knowledge, no previous study has applied a multi-level intervention approach to improve adherence to AHT [39]. Applying this approach to the cancer treatment and survivorship phases of the cancer control continuum is novel because most multi-level research in this area focuses on prevention and screening [26,40]. There is a similar pattern within the field of breast cancer research, where there are fewer intervention studies that focus on improving breast cancer treatment outcomes, compared to screening [41,42]. Among the studies that focus on improving adherence to AHT, few have found significant intervention effects [39,43-45] or are currently analyzing data [46].

In conclusion, achieving the proposed aims will contribute to the scientific community’s growing interest in multilevel intervention design and analyses; describe relationships between modifiable patient, family/social support, and health care system/team factors and adherence to AHT; and identify feasible intervention strategies for improving adherence to AHT among breast cancer survivors from racially and socioeconomically disadvantaged backgrounds.

## Appendix

Multimedia Appendix 1 NIH peer reviews.

## Abbreviations

AHT: Adjuvant hormonal therapy
GCCRI: Gibbs Cancer Center and Research Institute
HR+: Hormone receptor-positive
PI: Principal investigator
SCOA: South Carolina Oncology Associates
WHO: World Health Organization
