# Supplementary material for: Improving Adherence to Adjuvant Hormonal Therapy Among Disadvantaged Women Diagnosed with Breast Cancer in South Carolina: Proposal for a Multimethod Study
Source: JMIR Res Protoc. 2020 Sep 3;9(9):e17742. doi: 10.2196/17742 (PMC7499167; doi:10.2196/17742)
Supplement: Multimedia Appendix 1 [file resprot_v9i9e17742_app1.pdf]

SUMMARY STATEMENT
(Privileged Communication)

PROGRAM CONTACT:
Behrous Davani
240-276-6170
behrous.davani@nih.gov

Release Date: **11/19/2014**

Application Number: **1 K01 CA193667-01**

Principal Investigator

FELDER, TISHA

Applicant Organization: UNIVERSITY OF SOUTH CAROLINA AT COLUMBIA

Review Group: **NCI-I**
Subcommittee I - Transition to Independence

Meeting Date: **11/04/2014**
Council: **JAN 2015**
Requested Start: **04/01/2015**

RFA/PA: **PAR12-050**
PCC: **0FMB**

Project Title: **Improving Adherence to Adjuvant Hormonal Therapy among Disadvantaged Breast Cancer Survivors**
SRG Action: **Impact Score: [redacted]**
Next Steps: **Visit http://grants.nih.gov/grants/next_steps.htm**
Human Subjects: 30-Human subjects involved - Certified, no SRG concerns
Animal Subjects: 10-No live vertebrate animals involved for competing appl.
Gender: **1A-Both genders, scientifically acceptable**
Minority: **1A-Minorities and non-minorities, scientifically acceptable**
Children: **1A-Both Children and Adults, scientifically acceptable**
Clinical Research - not NIH-defined Phase III Trial

Project Year	Direct Costs Requested	Estimated Total Cost
1	[redacted]	[redacted]
2	[redacted]	[redacted]
3	[redacted]	[redacted]
4	[redacted]	[redacted]
5	[redacted]	[redacted]
	[redacted]	[redacted]
TOTAL	[redacted]	[redacted]

ADMINISTRATIVE BUDGET NOTE: The budget shown is the requested budget and has not been adjusted to reflect any recommendations made by reviewers. If an award is planned, the costs will be calculated by Institute grants management staff based on the recommendations outlined below in the COMMITTEE BUDGET RECOMMENDATIONS section.

1K01CA193667-01 Felder, Tisha

TRAINING IN THE RESPONSIBLE CONDUCT OF RESEARCH UNACCEPTABLE

RESUME AND SUMMARY OF DISCUSSION: In this new application for the NCI Mentored Research Scientist Development Award to Promote Diversity (K01), Dr. Felder proposes to develop and test a theory-based, multi-level intervention to improve adherence to adjuvant hormonal therapy (AHT) among breast cancer survivors from racial and socioeconomically-disadvantaged backgrounds. Dr. Felder is an outstanding applicant with an exceedingly strong background and experience in behavioral sciences. She is a recipient of numerous competitive awards and fellowships and has an excellent publication track record. The application is supported by outstanding letters of recommendation. Although the career development plan is in general well written, it could be further improved and optimized with regard to the proposed training and research activities and their timelines. Additional details regarding mentorship plans from some of the proposed mentors would also be helpful. The candidate has identified an outstanding team of established scientists clearly committed to supporting Dr. Felder's research and career development. The environment is outstanding in all respects and the institutional commitment to the candidate is adequate. The research plan is focused on a significant problem and supported by preliminary data generated by the candidate. However, some weaknesses were identified in the research plan. For example, some of the proposed studies are not described in sufficient detail and/or not adequately supported by the data provided. Specific aims seem to be dependent on each other and potential pitfalls and alternative approaches are not adequately addressed.

DESCRIPTION (provided by applicant): My long-term career goal is to become a leader in our nation's fight to eliminate disparities and improve health outcomes among racial/ethnic and socioeconomically-disadvantaged cancer survivors. Specifically, I desire to become a nationally-recognized outcomes researcher who: 1) develops and disseminates effective strategies to improve adherence to evidence-based cancer care; and 2) leads collaborative and productive transdisciplinary science that advances the field of cancer disparities research. I have substantial training in health services/outcomes research using secondary data analytic approaches (e.g., large administrative database analyses) to investigate disparities in pharmaceutical treatment use among disadvantaged populations. Training facilitated by this National Cancer Institute (NCI) Mentored Research Scientist Development Award to Promote Diversity (K01) will provide me with the protected time to obtain the theoretical knowledge and methodological skills necessary to achieve my long-term career goal. My training goals for this proposed K01 are to: a) develop skills in qualitative research methods; b) develop skills in the development and testing of behavioral interventions, including multi-level approaches; c) expand my knowledge of health communication; and d) increase my scholarly productivity in cancer disparities research. My mentors and I have developed a three-pronged strategy to achieve my career goals and ensure that my research training needs are addressed: 1) a strong, interdisciplinary team of mentors and collaborators who will guide my research and career development; 2) an innovative research project integrated with my training goals that is both scientifically relevant and rigorous in its design and methods; and 3) a schedule of didactic coursework, workshops, seminars and professional interactions that build upon the existing resources of the University of South Carolina and South Carolina's only NCI-designated cancer center—the Hollings Cancer Center of the Medical University of South Carolina. My proposed research plan seeks to address the paucity of existing intervention research focused on improving adherence to adjuvant hormonal therapy (AHT), particularly among racial and socioeconomically disadvantaged groups who experience excess rates of breast cancer mortality. Current clinical guidelines recommend hormone-receptor positive breast cancer survivors take AHT for at least 5 years. However, less than half of patients are adherent to guidelines and suboptimal AHT adherence is associated with increased risk of breast cancer mortality. Research has

extensively documented socio-demographic and disease-specific factors associated with adherence to AHT, but very little evidence exists on behavioral factors that can be modified (e.g., knowledge, patient-provider communication) and targeted by interventions. Identifying these modifiable intervention targets is critical to the development of efficacious strategies to improve adherence. Therefore, the overall goal of this study is to develop and test a theory-based, multi-level intervention to improve adherence to AHT among breast cancer survivors from racial and socioeconomically-disadvantaged backgrounds. The specific aims of this study are to: 1) explore multi- level (e.g., patient, healthcare system) factors that influence adherence to AHT; 2) develop a theory-based, multi-level intervention to improve adherence to AHT; and 3) pilot and evaluate a theory-based, multi-level intervention. The proposed studies will provide training, mentorship, and research experience that will provide the foundation for my career as an independent investigator committed to improving health outcomes and eliminating disparities among the growing cancer survivor population.

PUBLIC HEALTH RELEVANCE: The proposed research will address a significant gap in our knowledge of the modifiable factors that influence breast cancer survivors' adherence to adjuvant hormonal therapy (AHT), and will result in the development of a theory-based, multi-level intervention that may improve AHT adherence among racial/ethnic and socio- economically-disadvantaged (eg, Medicaid-insured) survivors who experience excess rates of breast cancer mortality. This intervention will be the first of its kind and has the potential to improve AHT adherence in an underserved population known for poor AHT adherence rates and for being disproportionately affected by breast cancer disparities.

The written critiques of individual reviewers are provided in essentially unedited form in this section. Please note that these critiques and criteria scores were prepared prior to the meeting and may not have been revised subsequent to any discussions at the review meeting. The "Resume and Summary of Discussion" section above summarizes the final opinions of the committee.

CRITIQUE 1:

Candidate: 4
Career Development Plan/Career Goals /Plan to Provide Mentoring: 3
Research Plan: 5
Mentor(s), Co-Mentor(s), Consultant(s), Collaborator(s): 4
Environment and Institutional Commitment to the Candidate: 4

Overall Impact:

This candidate proposes to develop and test a multi-level intervention to increase AHT adherence among disadvantaged patients. The candidate has considerable training in social policy, behavioral sciences, and outcome sciences. The candidate's limited record of first author research publications is a concern, as the plan for increased productivity is not adequate given other responsibilities and training. The career development plan is multidimensional and includes relevant training experiences, but the primary mentors are largely absent from training plan. There is minimal institutional support for the candidate without significant external support. An intervention like the one proposed may increase adherence, but the proposed data collection is inadequate. From the specific aims it is not clear whether the proposal is for disadvantaged populations or minorities; as such this research appears to overlap directly with Dr. Heiney's research and this is not adequately addressed. Adequate details of the data analyses are not provided, beyond mention of the software package. All research aims are dependent and this is also not adequately addressed. A letter attesting to the eligibility of the candidate is missing.

1. Candidate:

Strengths

- Candidate has MSW degree in Social policy and evaluation, PhD degree in Behavioral sciences, and several years of postdoctoral experience in outcomes sciences.
- Currently research assistant professor in College of Nursing at USC Columbia.
- Several research publications to date in the general area of disparities and cancer.

Weaknesses

- Limited first author research publications since 2011, despite full time research support.
- Current funding is unrelated to cancer or disadvantaged populations.
- Only one publication with previous postdoctoral advisor.

2. Career Development Plan/Career Goals & Objectives/Plan to Provide Mentoring:

Strengths

- Proposed career development does built on previous training and educational experience.
- Plan does include interactions with mentors, didactic training, and training for professional success.

Weaknesses

- The mentoring team is very large, and individuals with the most input to the training plan and the most relevant research background are not primary mentors.
- Some of the activities/timeline are not well motivated. For example, research aim 1 is scheduled for year 1 at which time the candidate will have one short course in qualitative data, but plans to develop an interview guide, coding, analysis, and interpretation of interview data with only monthly meetings with co-mentors (not primary mentors).
- There is limited input from the primary mentors in the training plan.
- The plan to increase scholarly productivity is unconvincing, particularly with the plethora of training activities proposed for years 1-3.
- A single cancer biology course in year 3 is insufficient.

3. Research Plan:

Strengths

- Multilevel multicomponent approach to intervention could address many factors related to medication adherence.
- Involvement of SCOA and Dr. Heiney in patient recruitment is very positive.

Weaknesses

- All three aims are dependent.
- Sample size is not adequately supported and very small given the multiple factors involved in adherence and the broad definition of breast cancer survivor. Insufficient data to support Aim 2.
- Insufficient consideration of pitfalls and limitations.

- Candidate states that studies show AHT significantly contributed to disparities in breast cancer mortality, but this is not adequately supported by her own research (see pg. 85).
- Research is not well motivated; previous interventions were not successful and it is not clear how the proposed plan will overcome this obstacle.
- Candidate suggests that pharmacists can lead intervention (see table 11.1) but a pharmacist is not involved in this proposal.

4. Mentor(s), Co-Mentor(s), Consultant(s), Collaborator(s):

Strengths

- Glowing letter of support from Mentors.
- The primary mentors are well established and well funded in their respective fields.

Weaknesses

- Dr. Felder's appointment is in the College of Nursing, but the primary mentors' are not.
- Dr. Skinner is not included in the letter of support, and her role appears to overlap with Dr. Ford. This confuses the distinction between consultants and collaborators.
- This proposal has significant overlap with Dr. Heiney's funded research; it is not clear why she does not have a larger role in this proposal.
- Dr. Hebert lists Dr. Felder as one of his K05 protégés, but Dr. Felder is currently supported to conduct non-cancer research.
- None of the previous trainees have completed their training and taken their research beyond the USC context.

5. Environment and Institutional Commitment to the Candidate:

Strengths

- Institution will allow Dr. Felder to commit 75% of her time to the proposed award.

Weaknesses

- Stated institutional resources are not being employed, such as statistical support. It is unclear what is meant by "other resources" that will be provided to support the proposed research as none are specifically mentioned.

Protections for Human Subjects:

Acceptable Risks and Adequate Protections

- acceptable risks and protections

Inclusion of Women, Minorities and Children:

- Sex/Gender: Distribution justified scientifically
- Race/Ethnicity: Distribution justified scientifically
- Inclusion/Exclusion of Children under 21: Excluding ages < 21 justified scientifically
- acceptable inclusion of women, minorities, and children

Vertebrate Animals:

Not Applicable (No Vertebrate Animals)

Biohazards:

Not Applicable (No Biohazards)

Training in the Responsible Conduct of Research:

Unacceptable

Comments on Format (Required):

- acceptable

Comments on Subject Matter (Required):

- acceptable

Comments on Faculty Participation (Required; not applicable for mid- and senior-career awards):

- not mentioned

Comments on Duration (Required):

- not mentioned

Comments on Frequency (Required):

- acceptable

Select Agents:

Not Applicable (No Select Agents)

Resource Sharing Plans:

Not Applicable (No Relevant Resources)

Budget and Period of Support:

Recommended budget modifications or possible overlap identified:

- Full time GRA is not justified. These activities should be conducted by the applicant as part of the proposed training.
- Travel to meet with Consultant is not justified.
- Laptops are not justified, as applicant is not dedicating 100% effort to this proposal.
- Funding for statistics licenses should be supported by stated institutional resources.
- NIH Regional Seminar on Program Funding and Grants Admin only requires a single attendance, not annually.
- Plan to compensate participants overvalues professionals and undervalues patient participation

CRITIQUE 2:

Candidate: 1
Career Development Plan/Career Goals /Plan to Provide Mentoring: 1
Research Plan: 3
Mentor(s), Co-Mentor(s), Consultant(s), Collaborator(s): 2
Environment and Institutional Commitment to the Candidate: 1

Overall Impact:

The candidate's career goal is to develop expertise in the area of disparities and health applied to cancer survivors. Short term goals via the K01 award are to develop knowledge in qualitative research methods, the development and testing of behavioral interventions and health communication while increasing productivity in cancer disparity research. The proposed research project for the K01 program pertains to intervention research to improve adherence to adjuvant hormonal therapy (AHT) among racial and socioeconomically disadvantaged groups with high rates of mortality from breast cancer. Strengths of the proposal are the excellent candidate, the impeccable training plan, the important and overall well-developed research project, the well-structured and competent group of mentors, collaborators and consultants, and the superb environment for the research program to be developed by the candidate. Minor weaknesses are found in the research plan that would require more details for some aspects.

1. Candidate:

Strengths

- The candidate was trained in health services/outcomes research using secondary analytic approaches with a focus on disparities in pharmaceutical treatment in disadvantaged populations. She has a BA degree in Sociology from Wake Forest University and a Master's in Social Work from University of Michigan in Social Policy & Evaluation, and a PhD degree in behavioral sciences from the UT School of Public Health and completed a postdoctoral training (2013) in the College of Pharmacy at USC.
- She received a constant string of awards linked to scholarly activities (including a highly competitive Presidential Management Fellow award to attract individuals to public service) since the late 90s and she is first author on six out of the 13 publications listed.
- She is a research assistant professor in the College of Nursing at USC.
- Excellent letters of support acknowledge the candidate's research accomplishments, her quest for constant improvement via advice from senior colleagues, her drive and commitment to research on health disparities and her high quality work. They describe the candidate as an ideal scientist for a K01 award.

Weaknesses

- None noted.

2. Career Development Plan/Career Goals & Objectives/Plan to Provide Mentoring:

Strengths

- The candidate clearly articulates the need for training in qualitative research methods, development of behavioral intervention and health communication as she spent training related to disparities so far on secondary analytic techniques. She also explains her journey towards health disparity research, notably via her exposure to NCI-related work early on and her

realization that the field of cancer disparities lacks minority researchers. Since then she has had rich and diverse training activities related to cancer disparities. She justifies well the need to learn how to develop and test multilevel interventions that reflect the involvement of patients, healthcare systems, etc. She greatly justifies the need for qualitative methods that provide context and meaning to complex phenomena and also enable the vulnerable populations to be heard.

- The training program is well organized and clearly presented with meeting schedules and role for each mentor.
- For each of the three career objectives the candidate explains what will be hands-on and research experience, interaction with mentors, shadowing clinicians and courses or workshops. Rationale is given for each aspect of the training and what the candidate will gain for these phases of the training. It is a very well organized training plan.
- Milestones are presented in terms of publications, presentations and grant applications with topics and expected years of achievement. Progress with the training plan will be evaluated by the mentoring committee four times per year; evaluation points are detailed. Cultural aspects of the training will be fulfilled via the candidate’s participation in the National Center for Faculty Development and Diversity’s Faculty Success Program.

Weaknesses

- None noted.

3. Research Plan:

The rationale for the proposed research is that there is almost no information on the behavioral factors that could be modified to improve adherence to AHT, thus impairing targeted intervention. The goal of the candidate is to develop and test a theory-based multi-level intervention to remedy low adherence to AHT. Aims are to (1) explore multi-level (patient, healthcare system, etc.) factors influencing adherence to AHT, (2) develop an intervention to improve adherence to AHT, and (3) evaluate this intervention.

Strengths

- The rationale and urgency to study factors that influence adherence to AHT in socioeconomically challenged populations in order to reduce disparity is well explained. The components and models to guide the project are identified. The candidate makes a point in justifying her choice for the specific study of AHT that goes beyond biologically-based differences among African American and Caucasian races. Choices for the different steps in the project are justified. The impact of this project on other interventions using medications is also discussed showing that the candidate has placed the specific analysis of adherence to AHT in the bigger context of intervention therapy with oral medications and survivorship. These aspects combined with the exploration of the impact of fashionable forms of communication also contribute to the innovative approach of the project.
- The proposed research is built on the candidate’s own preliminary data.
- Aim 1: the candidate will use in-depth semi-structured qualitative interviews with breast cancer survivors and healthcare professionals. Criteria for patients’ and healthcare personnel selections are clear and complete; the involvement of each collaborator to help with recruitment is indicated. The data analysis process is detailed.
- Aim 2: the candidate convincingly explains how models available and her attendance to courses such as “intervention mapping” as part of the training will inform decisions regarding the intervention design.

- Aim 3: this will be the pilot study for intervention that will be assessed rigorously via quasi-experimental, pre/posttest design (no control group). Criteria for recruitment of patient participants are clear, including the fact that they should be non-adherent to AHT.

Weaknesses

- Aim 1: The candidate does not adequately discuss how the type of drug used for AHT will be taken into account in the analysis. Yet, this might be an essential aspect of adherence to AHT since some of the drugs have very different levels of side effects and inconveniences that might differently impact adherence to their use.
- Aim 2: The candidate did not adequately explain how strongly the results from the patients compared to those obtained from the healthcare personnel will weigh on the design of the intervention. There might be a possibility that the healthcare 'point of views' undermine the information given by the patients. It is important that the candidate clearly describes her approach to reduce the risk of bias in her design from that standpoint. This is probably a question normally addressed in multi-level interventions but there is no adequate information regarding this issue in the proposal. Also the importance of the influence of family and friends in impacting the results should be adequately discussed.
- Aim 3: it is not clear what we will be the timeframe for the post-interview.
- The expectations, limitations and alternative section is not well developed; only one aspect is discussed: retention of participants. There are several other essential aspects to discuss, some of which were already suggested in the critique of the specific aims and others that pertain to more conceptual aspects of the project that, most likely, this competent candidate has in mind already. This section of the proposal is particularly important, since each aim is linked to the preceding one and thus, specific issues might profoundly affect the organization/conduct of the next aim.

4. Mentor(s), Co-Mentor(s), Consultant(s), Collaborator(s):

Strengths

- Co-primary mentor: Dr. Marvella Ford, Professor of biostatistics and epidemiology at the Medical University of South Carolina, Hollings Cancer Center. She is associate director of cancer disparities in the Cancer Center with the goal to increase the number of investigators in South Carolina who conduct research on cancer disparities. She has a record of external federal funding and a good list of publications. She is a behavioral scientist who has mentored 26 junior scientists (undergraduate to faculty level) since 2000. She will provide mentorship on intervention development in cancer disparity research.
- Co-primary mentor: Dr. James Hebert, professor of epidemiology and biostatistics and a Health Sciences Distinguished Professor, Arnold School of Public Health. He is the director of the USC Cancer Prevention and Control Program. He is committed to transdisciplinary and community-based participatory research (he has a K05 Established Investigator Award to develop this area of research). He has an extensive publication record (> 345 articles) and has an excellent track record of external funding. He will provide mentorship on intervention development and cancer epidemiology. He has described his mentorship track record and has provided information on his mentorship plan for the candidate and has listed her as one of the protégé on his K05 grant.
- Co-mentor: Dr. Ronit Elk, research associate professor in the college of Nursing. She is an expert in health policy and disparities and has worked for 10 years to build the American Cancer Society program in health disparities before coming back to academia (since 2012). She will provide mentorship on career development.

- Co-mentor: Dr. Daniela Friedman, Associate Professor of Health Promotion, Education & Behavior, Arnold School of Public Health. She is PI and co-PI on several projects related to dissemination, implementation and evaluation of evidence-based cancer prevention and control interventions. She lists a K01 awardee as a current mentee in addition to a few graduate students. She has an excellent publication track record. She will provide mentorship on qualitative research and health communication.
- Co-mentor: Dr. Sue Heiney, Dunn-Shealy Research Professor in the College of Nursing, is the co-director of the Cancer Survivorship Center at USC. She has expertise in intervention studies with group approaches. She will provide mentorship on qualitative research, intervention development and study recruitment.
- Collaborators and consultants will provide help with biostatistics, breast oncology care and multilevel intervention development.
- The candidate has support letters from the president of the South Carolina Oncology Associates (to help recruit low-income individuals for the project) and from a consultant from UT Southwestern on multi-level initiatives in cancer care.

Weaknesses

- Drs. Ford and Elk did not give sufficient details regarding the mentorship plan for the candidate.
- It might have been helpful to have biosketches of the collaborators and consultant since they will participate in the training of the candidate.

5. Environment and Institutional Commitment to the Candidate:

Strengths

- The environment at the University of South Carolina (USC) is excellent, commended by the Carnegie Foundation for “very high research activity”, the only university in South Carolina in this category. It will provide access for the candidate to resources and facilities there and also at the NCI-designated Hollings Cancer Center since the candidate is an affiliate member of the cancer control program.
- The college of nursing works in collaboration with other health disciplines (pharmacy, social work, public health, medicine) for health planning, grant writing, delivering services and developing educational programs.
- The Cancer Survivorship Center will provide a network of clinical and academic experts available to the candidate for her research.
- The candidate is also a core-faculty affiliate of the Statewide Cancer Prevention and Control Program housed in the School of Public Health that focuses on large cancer health disparities in African American populations.
- There are also other centers focusing on health disparity in South Carolina, including the Institute for Partnerships to Eliminate Health Disparities and the South Carolina Rural Health Research Center, as well as the Palmetto Health not-for-profit healthcare system that is dedicated to working with individuals and centers to fight against health disparities.
- The institutional letter of support is excellent; it indicates protected time as requested but also support towards a tenure track position based on external funding and scholarly publications.

Weaknesses

- None noted.

Protections for Human Subjects:

Acceptable Risks and Adequate Protections

- all necessary aspects are detailed

Inclusion of Women, Minorities and Children:

- Sex/Gender: Distribution justified scientifically
- Race/Ethnicity: Distribution justified scientifically
- Inclusion/Exclusion of Children under 21: Including ages < 21 justified scientifically

Vertebrate Animals:

Not Applicable (No Vertebrate Animals)

Biohazards:

Not Applicable (No Biohazards)

Training in the Responsible Conduct of Research:

Acceptable

Comments on Format (Required):

- courses, online readings, modules, workshop

Comments on Subject Matter (Required):

- major subject matters pertaining to the candidate's line of research are listed.

Comments on Faculty Participation (Required; not applicable for mid- and senior-career awards):

- acceptable

Comments on Duration (Required):

- at least four years

Comments on Frequency (Required):

- a different program every year for years 1 to 4, the frequency for each activity is not detailed.

Select Agents:

Not Applicable (No Select Agents)

Budget and Period of Support:

Recommend as Requested

Recommended budget modifications or possible overlap identified:

- The budget justification does not seem to match the yearly forms; also there is no information on the FTE for a graduate student; based on the amount budgeted it does not seem to correspond to a full stipend.

Additional Comments to Applicant (Optional):

- Very well written and interesting proposal.

CRITIQUE 3:

Candidate: 1
Career Development Plan/Career Goals /Plan to Provide Mentoring: 1
Research Plan: 5
Mentor(s), Co-Mentor(s), Consultant(s), Collaborator(s): 3
Environment and Institutional Commitment to the Candidate: 1

Overall Impact:

This is a new application from an African American behavioral scientist who is a Research Assistant Professor at the University of South Carolina School of Nursing/School of Public Health. The strengths of the application are the Candidate who has clearly dedicated herself to the study of disparities in cancer treatment, a strong Mentoring team, and an outstanding Career Development Plan. The subject of adherence to adjuvant hormonal therapy in breast cancer is highly significant, but the lack of an explicit hypothesis is considered at least a moderate weakness in the Research Plan.

1. Candidate:

Strengths

- Background includes MSW degree with part-time work as social worker in Detroit prior to research career.
- Presidential Management Fellowship at the NCI 2003-2005 started academic career.
- PhD degree in Behavioral Sciences from University of Texas School of Public Health in 2010 on topic of pharmaceutical assistance programs.
- Post-doctoral training at MUSC funded by Pharmaceutical Research and Manufacturers of America Foundation, further experience with secondary analysis of large datasets.
- 11 publications, including 6 first author ones. Most recent was review/editorial in 2013 in J Onc Practice on cost of oral cancer drugs.
- Track record of internal and foundation funding.

Weaknesses

- None noted.

2. Career Development Plan/Career Goals & Objectives/Plan to Provide Mentoring:

Strengths

- Clearly defined new areas of development in qualitative research, behavioral intervention development, and health communication.
- Exemplary career development plan - well-organized, detailed, including frequency of interactions and specific role of mentors/advisors in the project.

Weaknesses

- None noted.

3. Research Plan:

Strengths

- There are large racial disparities in breast cancer so this is a significant problem, and South Carolina seems to be an ideal state to study this problem with greater than average disparities .
- There are compelling data that adherence to adjuvant hormonal therapy (AHT) improves survival in HR+ breast cancers and that this may contribute to racial disparities.

Weaknesses

- There is not an explicitly-stated hypothesis. For example, is it expected that lack of adherence is most commonly due to socioeconomic factors?
- Candidate's own prior data demonstrate that among Medicaid patients there is no difference in adherence based on race. Does limiting this study to Medicaid patients perhaps miss important non-socioeconomic factors that may contribute to racial disparities?

4. Mentor(s), Co-Mentor(s), Consultant(s), Collaborator(s):

Strengths

- Primary Mentor, Dr. Hebert (at USC) is an epidemiologist and Director of SC's Cancer Prevention and Control Program and is expert in field of disparities. He has U54 and K05.
- Co-Primary Mentor, Dr. Ford (at MUSC) is a behavior scientist with expertise in interventions to reduce disparities and track record of mentoring minority trainees.
- Three additional "co-mentors", Drs. Friedman, Elk, Heiney plus three collaborators/consultants provide additional expertise, some seems complementary, some may be redundant, but overall it is a strong team with a very strong "team" letter of support for Candidate.

Weaknesses

- Charleston is not that close to Columbia (2-3 hours drive). She clearly describes regular meetings with both her USC and MUSC mentors/co-mentors. Some acknowledgment of this geographic limitation is expected, and clarification as to whether she will be commuting back and forth or whether some meetings will be by phone.

5. Environment and Institutional Commitment to the Candidate:

Strengths

- Currently in pre-tenure track position but with commitment to transition to tenure track with receipt of extramural funding.

Weaknesses

- None noted.

Protections for Human Subjects:

Acceptable Risks and Adequate Protections

Inclusion of Women, Minorities and Children:

- Sex/Gender: Distribution justified scientifically
- Race/Ethnicity: Distribution justified scientifically
- Inclusion/Exclusion of Children under 21: Including ages < 21 justified scientifically

Vertebrate Animals:

Not Applicable (No Vertebrate Animals)

Training in the Responsible Conduct of Research:

Acceptable

Budget and Period of Support:

Recommend as Requested

THE FOLLOWING SECTIONS WERE PREPARED BY THE SCIENTIFIC REVIEW OFFICER TO SUMMARIZE THE OUTCOME OF DISCUSSIONS OF THE REVIEW COMMITTEE, OR REVIEWER'S WRITTEN CRITIQUES, ON THE FOLLOWING ISSUES:

PROTECTION OF HUMAN SUBJECTS (Resume): ACCEPTABLE

INCLUSION OF WOMEN PLAN (Resume): ACCEPTABLE

INCLUSION OF MINORITIES PLAN (Resume): ACCEPTABLE

INCLUSION OF CHILDREN PLAN (Resume): ACCEPTABLE

TRAINING IN THE RESPONSIBLE CONDUCT OF RESEARCH: UNACCEPTABLE

COMMITTEE BUDGET RECOMMENDATIONS:
The budget was recommended as requested. However, the justification for the budget was insufficiently clear or inadequately justified in some aspects and it is suggested that NCI staff review the budget request and make adjustments as needed.

NIH has modified its policy regarding the receipt of resubmissions (amended applications). See Guide Notice NOT-OD-14-074 at http://grants.nih.gov/grants/guide/notice-files/NOT-OD-14-074.html. The impact/priority score is calculated after discussion of an application by averaging the overall scores (1-9) given by all voting reviewers on the committee and multiplying by 10. The criterion scores are submitted prior to the meeting by the individual reviewers assigned to an application, and are not discussed specifically at the review meeting

or calculated into the overall impact score. Some applications also receive a percentile ranking. For details on the review process, see http://grants.nih.gov/grants/peer_review_process.htm#scoring.

MEETING ROSTER

Subcommittee I - Transition to Independence
National Cancer Institute Initial Review Group
NATIONAL CANCER INSTITUTE
NCI-I
November 04, 2014 - November 05, 2014

CHAIRPERSON

OSHEROFF, NEIL , PHD
PROFESSOR OF BIOCHEMISTRY AND MEDICINE, JOHN G. CONIGLIO CHAIR IN BIOCHEMISTRY
DEPARTMENT OF BIOCHEMISTRY
VANDERBILT UNIVERSITY SCHOOL OF MEDICINE
NASHVILLE, TN 372320146

MEMBERS

AKPORIAYE, EMMANUEL T., PHD
PROFESSOR AND DIVISION CHIEF OF EXPERIMENTAL BIOLOGY
LABORATORY OF TUMOR IMMUNOLOGY & THERAPEUTICS
ROBERT W. FRANZ CANCER RESEARCH CENTER
EARLE A. CHILES RESEARCH INSTITUTE
PROVIDENCE PORTLAND MEDICAL CENTER
PORTLAND, OR 97213

AYER, DONALD E., PHD *
PROFESSOR
DEPARTMENT OF ONCOLOGICAL SCIENCES
HUNTSMAN CANCER INSTITUTE
UNIVERSITY OF UTAH
SALT LAKE CITY, UT 84112

BOISE, LAWRENCE H., PHD
PROFESSOR
DEPARTMENT OF HEMATOLOGY & MEDICAL ONCOLOGY
WINSHIP CANCER INSTITUTE
EMORY UNIVERSITY SCHOOL OF MEDICINE
ATLANTA, GA 30322

BOUTON, AMY H., PHD
PROFESSOR, ASSOCIATE DEAN FOR GRADUATE AND MEDICAL SCIENTIST PROGRAMS
DEPARTMENT OF MICROBIOLOGY, IMMUNOLOGY AND CANCER BIOLOGY
UNIVERSITY OF VIRGINIA SCHOOL OF MEDICINE
CHARLOTTESVILLE, VA 22908

BURNETT, ANDREAN L., PHD *
ASSISTANT PROFESSOR
DEPARTMENT OF PATHOLOGY
THE UNIVERSITY OF IOWA
IOWA CITY, IA 52242

BYERS, STEPHEN W., PHD *
PROFESSOR AND ASSOCIATE DIRECTOR
DEPARTMENT OF ONCOLOGY
LOMBARDI CANCER CENTER
GEORGETOWN UNIVERSITY
WASHINGTON, DC 20007

CALIFANO, JOSEPH A. III, MD *
PROFESSOR
DEPARTMENT OF OTOLARYNGOLOGY
HEAD AND NECK SURGERY
JOHNS HOPKINS UNIVERSITY
BALTIMORE, MD 21205

CARLESSO, NADIA , MD, PHD *
ASSOCIATE PROFESSOR
DEPARTMENT OF PEDIATRICS
SCHOOL OF MEDICINE
INDIANA UNIVERSITY
INDIANAPOLIS, IN 46202

FENG, ZHAOHUI , PHD *
ASSOCIATE PROFESSOR
DEPARTMENT OF RADIATION ONCOLOGY & PHARMACOLOGY
THE CANCER INSTITUTE OF NEW JERSEY
RUTGERS, STATE UNIVERSITY OF NEW JERSEY
ROBERT WOOD JOHNSON MEDICAL SCHOOL
NEW BRUNSWICK, NJ 08901

GOGA, ANDREI , MD, PHD
ASSOCIATE PROFESSOR
CELL & TISSUE BIOLOGY
DEPARTMENT OF MEDICINE
SCHOOL OF DENTISTRY AND MEDICINE
UNIVERSITY OF CALIFORNIA, SAN FRANCISCO
SAN FRANCISCO, CA 94143

GRAVES, EDWARD E., PHD *
ASSOCIATE PROFESSOR
DEPARTMENT OF RADIATION ONCOLOGY
MOLECULAR IMAGING PROGRAM AT STANFORD
STANFORD UNIVERSITY
STANFORD, CA 94305

HAWKINS, WILLIAM G., MD *
ASSOCIATE PROFESSOR
DEPARTMENT OF SURGERY
WASHINGTON UNIVERSITY SCHOOL OF MEDICINE
ST. LOUIS, MO 63110

HSIEH, JER-TSONG , PHD *
PROFESSOR AND DISTINGUISHED CHAIR IN PROSTATE CANCER RESEARCH
DEPARTMENT OF UROLOGY
UNIVERSITY OF TEXAS SOUTHWESTERN MEDICAL CENTER
DALLAS, TX 75390

JERUSS, JACQUELINE S., MD, PHD *
ASSOCIATE PROFESSOR
DEPARTMENT OF SURGERY
UNIVERSITY OF MICHIGAN
ANN ARBOR, MI 481035932

JIANG, YU , PHD *
ASSOCIATE PROFESSOR
DEPARTMENT OF PHARMACOLOGY
AND CHEMICAL BIOLOGY
UNIVERSITY OF PITTSBURGH
PITTSBURGH, PA 15261

KELLER, CHARLES , MD
ASSOCIATE PROFESSOR
DEPARTMENT OF PEDIATRICS
PEDIATRIC CANCER BIOLOGY PROGRAM
PAPE' FAMILY PEDIATRIC RESEARCH INSTITUTE
OREGON HEALTH & SCIENCE UNIVERSITY
PORTLAND, OR 972393098

KOO, JA SEOK , PHD *
ASSOCIATE PROFESSOR
YALE CANCER CENTER
YALE SCHOOL OF MEDICINE
NEW HAVEN, CT 06520

KROHN, KENNETH A., PHD
PROFESSOR OF RADIOLOGY AND RADIATION ONCOLOGY
ADJUNCT PROFESSOR OF CHEMISTRY
ASSOCIATE PROGRAM DIRECTOR, CANCER IMAGING PROGRAM
UNIVERSITY OF WASHINGTON
SEATTLE, WA 98195

LELIEVRE, SOPHIE A., DVM, PHD, LLMPH
ASSOCIATE PROFESSOR
ASSOCIATE DIRECTOR, DISCOVERY GROUPS
PURDUE CENTER FOR CANCER RESEARCH
ONCOLOGICAL SCIENCES CENTER INVESTIGATOR
PURDUE UNIVERSITY
WEST LAFAYETTE, IN 479072060

LIANG, CHENGYU , MD, PHD *
ASSISTANT PROFESSOR
KECK SCHOOL OF MEDICINE
UNIVERSITY OF SOUTHERN CALIFORNIA
LOS ANGELES, CA 9033

LOKSHIN, ANNA E., PHD
PROFESSOR
DEPARTMENT OF MEDICINE, PATHOLOGY,
AND OB/GYN RS
UPMC HILLMAN CANCER CENTER
UNIVERSITY OF PITTSBURGH SCHOOL OF MEDICINE
PITTSBURGH, PA 15213

MANNE, UPENDER , PHD *
PROFESSOR OF PATHOLOGY, SENIOR SCIENTIST,
COMPREHENSIVE CANCER CENTER, SENIOR SCIENTIST,
MINORITY HEALTH & HEALTH DISPARITIES RESEARCH CENTER
WALLACE TUMOR INSTITUTE
COMPREHENSIVE CANCER CENTER
UNIVERSITY OF ALABAMA AT BIRMINGHAM
BIRMINGHAM, AL 35294

MATEI, DANIELA E., MD
ASSOCIATE PROFESSOR
DEPARTMENT OF MEDICINE; HEMATOLOGY-ONCOLOGY
CO-LEADER EXPERIMENTAL AND DEVELOPMENTAL THERAPEUTICS PROGRAM
INDIANA UNIVERSITY SCHOOL OF MEDICINE
INDIANAPOLIS, IN 46202

NONN, LARISA , PHD *
ASSISTANT PROFESSOR
DEPARTMENT OF PATHOLOGY
UNIVERSITY OF ILLINOIS AT CHICAGO
CHICAGO, IL 60612

PAULSEN, KEITH D., PHD
ROBERT A. PRITZER PROFESSOR OF BIOMEDICAL ENGINEERING
DIRECTOR, DARTMOUTH ADVANCED IMAGING CENTER
DEPARTMENT OF BIOMEDICAL ENGINEERING AND RADIOLOGY
THAYER SCHOOL OF ENGINEERING
DARTMOUTH COLLEGE
HANOVER, NH 03755

SARKAR, DEVANAND , PHD *
ASSOCIATE PROFESSOR
DEPARTMENT OF HUMAN AND MOLECULAR GENETICS
HARRISON ENDOWED SCHOLAR IN CANCER RESEARCH
MASSEY CANCER CENTER
VIRGINIA COMMONWEALTH UNIVERSITY
RICHMOND, VA 23298

SAUSVILLE, EDWARD A., FACP, MD, PHD
PROFESSOR OF MEDICINE, DEPUTY DIRECTOR
MARLENE AND STEWART GREENEBAUM CANCER CENTER
DEPARTMENT OF MEDICINE
UNIVERSITY OF MARYLAND SCHOOL OF MEDICINE
BALTIMORE, MD 21201

SEAGROVES, TIFFANY N., PHD
ASSOCIATE PROFESSOR
DIRECTOR, GERWIN SMALL ANIMAL IMAGING CENTER, CRB
CO-TRACK HEAD, CANCER AND DEVELOPMENTAL BIOLOGY TRACK, IBS PROGRAM
UNIVERSITY OF TENNESSEE HEALTH SCIENCE CENTER
MEMPHIS, TN 38163

SOLIT, DAVID B., MD
ASSOCIATE MEMBER
DEPARTMENT OF MEDICINE
MEMORIAL SLOAN-KETTERING CANCER CENTER
WEILL CORNELL MEDICAL COLLEGE
NEW YORK, NY 10065

TAN, MING T., PHD *
PROFESSOR AND CHAIR
DEPARTMENT OF BIOSTATISTICS, BIOINFORMATICS & BIOMATHEMATICS
MEDICAL CENTER
GEORGETOWN UNIVERSITY
WASHINGTON, DC 20057

TANNOUS, BAKHOS A., PHD
ASSOCIATE PROFESSOR OF NEUROLOGY
MASSACHUSETTS GENERAL HOSPITAL
HARVARD MEDICAL SCHOOL
CHARLESTOWN, MA 02129

THOMAS-TIKHONENKO, ANDREI , PHD *
PROFESSOR AND DIVISION CHIEF
DIVISION OF CANCER PATHOBIOLOGY
THE CHILDREN'S HOSPITAL OF PHILADELPHIA
PERELMAN SCHOOL OF MEDICINE AT THE
UNIVERSITY OF PENNSYLVANIA
PHILADELPHIA, PA 191044399

THOMPSON, E. AUBREY, PHD
PROFESSOR OF CANCER BIOLOGY
DEPARTMENTS OF BIOCHEMISTRY, MOLECULAR
BIOLOGY,
AND CANCER BASIC SCIENCE
MAYO CLINIC COMPREHENSIVE CANCER CENTER
JACKSONVILLE, FL 32224

WANG, SHIZHEN EMILY, PHD
ASSOCIATE PROFESSOR
DEPARTMENT OF CANCER BIOLOGY
BECKMAN RESEARCH INSTITUTE OF CITY OF HOPE
DUARTE, CA 91010

WHITE, REBEKAH , MD *
ASSOCIATE PROFESSOR
DEPARTMENT OF SURGERY
DUKE UNIVERSITY MEDICAL CENTER
DURHAM, NC 27710

YANG, YU-CHUNG , PHD
PROFESSOR
DEPARTMENT OF BIOCHEMISTRY
CASE WESTERN RESERVE UNIVERSITY
SCHOOL OF MEDICINE
CLEVELAND, OH 44106

MAIL REVIEWER(S)

CLARKE, JENNIFER P., PHD
DIRECTOR, COMPUTATIONAL SCIENCES INITIATIVE,
ASSOCIATE PROFESSOR, DEPARTMENT OF FOOD
SCIENCE AND TECHNOLOGY
DEPARTMENT OF FOOD SCIENCE TECHNOLOGY
UNIVERSITY OF NEBRASKA, LINCOLN
LINCOLN, NE 68583

ZU, YOULI , MD, PHD
MEDICAL DIRECTOR, HEMATOPATHOLOGY
DEPARTMENT OF PATHOLOGY AND GENOMIC MEDICINE
THE METHODIST HOSPITAL
WEILL MEDICAL COLLEGE OF CORNELL UNIVERSITY
HOUSTON, TX 77030

SCIENTIFIC REVIEW OFFICER

RADAEV, SERGEI , PHD
SCIENTIFIC REVIEW OFFICER
RESOURCES AND TRAINING REVIEW BRANCH
DIVISION OF EXTRAMURAL ACTIVITIES
NATIONAL CANCER INSITUTE
NATIONAL INSTITUTES OF HEALTH
BETHESDA, MD 20892

EXTRAMURAL SUPPORT ASSISTANT

HOLLOWAY, GELIA
LEAD EXTRAMURAL SUPPORT ASSISTANT
RESOURCES AND TRAINING REVIEW BRANCH
DIVISION OF EXTRAMURAL ACTIVITIES SUPPORT
NATIONAL CANCER INSTITUTE
NATIONAL INSTITUTES OF HEALTH
BETHESDA, MD 20892

PROGRAM REPRESENTATIVE

JAKOWLEW, SONIA B, PHD
PROGRAM DIRECTOR
CANCER TRAINING BRANCH
CENTER FOR CANCER TRAINING
NATIONAL CANCER INSTITUTE
NATIONAL INSTITUTES OF HEALTH
BETHESDA, MD 20892

OJEIFO, JOHN O, PHD, MBBS
PROGRAM DIRECTOR
DIVERSITY TRAINING BRANCH
CENTER TO REDUCE CANCER HEALTH DISPARITIES
NATIONAL CANCER INSTITUTE
NATIONAL INSTITUTES OF HEALTH
BETHESDA, MD 20892

SCHMIDT, MICHAEL K, PHD
PROGRAM DIRECTOR
CANCER TRAINING BRANCH,
CENTER FOR CANCER TRAINING
NATIONAL CANCER INSTITUTE, NIH
ROCKVILLE, MD 20852

WALI, ANIL , PHD
PROGRAM DIRECTOR
CENTER TO REDUCE CANCER HEALTH DISPARITIES
NATIONAL CANCER INSTITUTE
NATIONAL INSTITUTE OF HEALTH
BETHESDA, MD 20852

* Temporary Member. For grant applications, temporary members may participate in the entire meeting or may review only selected applications as needed.

Consultants are required to absent themselves from the room during the review of any application if their presence would constitute or appear to constitute a conflict of interest.
